# Does Publication Bias Inflate the Apparent Efficacy of Psychological Treatment for Major Depressive Disorder? A Systematic Review and Meta-Analysis of US National Institutes of Health-Funded Trials

**DOI:** 10.1371/journal.pone.0137864

**Published:** 2015-09-30

**Authors:** Ellen Driessen, Steven D. Hollon, Claudi L. H. Bockting, Pim Cuijpers, Erick H. Turner

**Affiliations:** 1 Department of Clinical, Neuro and Developmental Psychology, Faculty of Behavioural and Movement Sciences, VU University Amsterdam, Amsterdam, The Netherlands; 2 EMGO Institute for Health and Care Research, VU University and VU University Medical Center Amsterdam, Amsterdam, The Netherlands; 3 Department of Psychology, Vanderbilt University, Nashville, Tennessee, United States of America; 4 Department of Clinical Psychology, University of Groningen, Groningen, The Netherlands; 5 Department of Clinical and Health Psychology, Utrecht University, Utrecht, The Netherlands; 6 Behavioral Health and Neurosciences Division, VA Portland Health Care System, Portland, Oregon, United States of America; 7 Departments of Psychiatry and Pharmacology, Oregon Health & Science University, Portland, Oregon, United States of America; Peking University, CHINA

## Abstract

**Background:**

The efficacy of antidepressant medication has been shown empirically to be overestimated due to publication bias, but this has only been inferred statistically with regard to psychological treatment for depression. We assessed directly the extent of study publication bias in trials examining the efficacy of psychological treatment for depression.

**Methods and Findings:**

We identified US National Institutes of Health grants awarded to fund randomized clinical trials comparing psychological treatment to control conditions or other treatments in patients diagnosed with major depressive disorder for the period 1972–2008, and we determined whether those grants led to publications. For studies that were not published, data were requested from investigators and included in the meta-analyses. Thirteen (23.6%) of the 55 funded grants that began trials did not result in publications, and two others never started. Among comparisons to control conditions, adding unpublished studies (Hedges’ *g* = 0.20; CI_95%_ -0.11~0.51; k = 6) to published studies (*g* = 0.52; 0.37~0.68; k = 20) reduced the psychotherapy effect size point estimate (*g* = 0.39; 0.08~0.70) by 25%. Moreover, these findings may overestimate the "true" effect of psychological treatment for depression as outcome reporting bias could not be examined quantitatively.

**Conclusion:**

The efficacy of psychological interventions for depression has been overestimated in the published literature, just as it has been for pharmacotherapy. Both are efficacious but not to the extent that the published literature would suggest. Funding agencies and journals should archive both original protocols and raw data from treatment trials to allow the detection and correction of outcome reporting bias. Clinicians, guidelines developers, and decision makers should be aware that the published literature overestimates the effects of the predominant treatments for depression.

## Introduction

Publication bias has been defined as the tendency for authors to submit, or journals to accept, manuscripts for publication based on the direction or strength of the study’s findings [1]. It has been long recognized that publication bias can lead to an overestimation of treatment effects [2] that threatens the validity of evidence-based decisions.

Major depression is a highly prevalent and disabling disorder associated with major personal and societal costs [3–5]. It is the fourth leading cause of disease burden worldwide and is expected to rank first in high-income countries by the year 2030 [6]. Antidepressant medication is recommended as a first-line treatment for major depressive disorder in most treatment guidelines [7–8] and the majority of depressed patients are now so treated in primary care [9]. However, the efficacy of pharmacotherapy for depression has been overestimated due to publication bias. Turner and colleagues [10] found that publication was strongly linked to outcome in 74 placebo-controlled antidepressant studies submitted to the US Food and Drug Administration (FDA). Among 38 studies judged positive by the FDA, all but one were published in a way that agreed with those judgments. By contrast, among 36 studies judged negative or questionable by the FDA, all but 3 were subjected to publication bias in one of two forms: (1) the studies were not published (study publication bias, 61%), or (2) negative results were “spun” to make them look positive (outcome reporting bias, 31%) through selective reporting of sites, subjects (especially dropouts), and measures. Comparing the published literature with the original FDA data resulted in a 24% reduction in pooled mean effect size for pharmacotherapy versus pill-placebo, from 0.41 (CI_95%_ 0.36~0.45) to 0.31 (0.27~0.35).

The above-mentioned treatment guidelines also recommend the use of a depression-focused psychotherapy as an alternative to medications for patients with mild to moderate major depressive disorder [7–8]. However, one might ask whether the effects of psychological treatments for depression could also be overestimated due to publication bias. Cuijpers and colleagues [11] examined 117 published trials, including 175 comparisons between psychological treatments and control conditions, and using statistical procedures [12–13], found evidence for 51 “missing” studies; when their results were imputed, the overall effect size was reduced by 37%, from 0.67 (CI_95%_ 0.60~0.75) to 0.42 (0.33~0.51).

This suggests that psychological treatment, like pharmacologic treatment, may not be as efficacious as the published literature would indicate. However, the statistical procedures used by Cuijpers et al. [11] relied on the assumption that any asymmetry in the funnel plots, which depict the association between sample size and effect size in meta-analyses, stems from a failure to publish small studies with small effect sizes. Nonetheless, small studies may show disproportionately large effects for reasons other than publication bias [14–15]. Thus, as Borenstein and colleagues [15] state: “the only true test for publication bias is to compare effects in the published studies formally with effects in the unpublished studies” (p. 280).

In the present study, we sought to estimate the “true” effect of study publication bias by examining a cohort of trials funded by the US National Institutes of Health (NIH). The NIH is an agency of the US Department of Health and Human Services and is the primary agency of the United States government responsible for biomedical and health-related research, in which it invests over $30.9 billion annually. The majority of NIH funding is awarded through competitive grants to researchers at universities, medical schools, and other research institutions in the US and abroad. We identified grants awarded for randomized clinical trials examining the efficacy of psychological treatments, compared to control or alternative interventions, in adult or geriatric patients with major depressive disorder. We ascertained the frequency with which these studies were conducted but not published. In addition, we sought to better estimate the effect of psychological treatment on major depressive disorder by pooling unpublished with published findings.

## Methods

No review protocol exists beyond what is described in our methods section.

### Defining the set of relevant funded grants

We searched NIH databases (see below) for grants, funding randomized clinical trials testing the effects of psychological treatments on major depressive disorder for the years 1972 through May 2008. NIH records do not go back before 1972; and the cutoff date was established in order to allow at least 60 months (5 years) for publication of trial results before our search started in June 2013. This duration was derived from a study of time to publication among 635 NIH-funded clinical trials, which showed that about 95% of to-be-published trials were published within 60 months of trial completion [16].

We used http://crisp.cit.nih.gov/crisp_query.generate_screen to search for relevant grants through 1984 and http://projectreporter.nih.gov/reporter.cfm for trials completed from 1985 on. These databases provide summary information on the grants and often include abstracts. We searched using all possible combinations of terms for (1) “depression” (depression, depressive, major depressive disorder, mood disorder, affective disorder, melancholic, melancholia) and (2) “psychological treatment” (cognitive therapy, behavior therapy, behavioral therapy, interpersonal therapy, psychodynamic therapy, dynamic therapy, humanistic therapy, therapy, supportive therapy, experiential therapy, [self-] control therapy, [problem-] solving therapy, [supportive-] expressive therapy, family therapy, group therapy, marital therapy, couples therapy, aversive therapy, exposure therapy, psychotherapy, psychotherapies, psychotherapeutic, counseling, disease management, psychoanalytic, behavioral activation, cognitive behavioral analysis system, desensitization, relaxation techniques, and progressive muscle relaxation). We deleted duplicate listings and multiple years for the same grants.

We then screened the grant titles and available abstracts for possible inclusion. We followed the methods used by Turner and colleagues [10] as closely as possible in order to facilitate comparison of our results to those with pharmacological trials. We included all grants that proposed to conduct (1) a randomized clinical trial examining (2) psychological treatment for (3) acute depression in (4) adults. We used measures of depressive symptoms as the sole outcome for this meta-analysis. Following Cuijpers and colleagues [11], we defined psychological treatment as any intervention in which verbal communication between therapist and client was the core element. We also included bibliotherapy if it was supplemented with personal support from a therapist. We included treatments aimed at decreasing depressive symptoms and excluded treatments aimed solely at increasing adherence to antidepressant medication. We only included studies in which the differential efficacy of psychological treatment could be ascertained. Thus, we included studies that examined the efficacy of psychological treatment combined with antidepressant medication versus that of medication alone, but we excluded studies in which all patients received the same psychological treatment and studies in which not all patients in a condition received psychological treatment.

Depression was defined as meeting diagnostic criteria for major depressive disorder (MDD) or its equivalent in an earlier nosology (e.g., Research Diagnostic Criteria [17] or Feighner criteria [18]). We included studies that sampled patients who did not meet criteria for MDD only if outcomes were reported separately for those who did (since those were the patients included in our analyses). We excluded studies on children and adolescents younger than 18, but we included studies with geriatric patients since more recent studies of adults and elderly patients have found that psychological treatment efficacy does not differ with age [19].

### Matching grants to published articles

We consulted www.evidencebasedpsychotherapies.org, a publicly available database of 352 randomized clinical trials regarding psychological treatment for adult depression [20], in order to identify published articles. This database is updated annually through comprehensive literature searches in Pubmed, PsycINFO, Embase and the Cochrane Central Register of Controlled Trials. We consider this database as defining the published literature on randomized clinical trials for the psychological treatment of depression.

We used this database as one source for matching published articles to the grants. We matched a published article to a grant based on the grant number acknowledged in the paper, making sure that it also matched with respect to investigator, type of psychological treatment, and comparator. When we could not find a matching publication within the database, we searched PubMed for all articles published by the principal investigator. If we still could not find a matching publication, we contacted the investigator to ask whether those findings had ever been published.

We included articles that reported outcomes for the short-term treatment of depression; we excluded articles reporting the prevention of subsequent relapse (continuation treatment) or recurrence (maintenance treatment). When multiple sequential articles were found reporting acute response on the same outcomes, we selected the one that was most complete in terms of sample size. We excluded articles that reported secondary analyses in favor of articles reporting primary data.

In order to ensure that we had not missed relevant grants with our grant search strategy, we checked all the published studies in the database just described to see if they acknowledged NIH grant funding. Whenever we found an article that acknowledged a grant that was not in our list, we resolved the discrepancy, if necessary by contacting the principle investigator. All published articles matched to the NIH grants were read independently by two authors to ensure that they met inclusion criteria. Disagreements were resolved by consensus.

### Assessment of study publication bias

We calculated: (1) the proportion of funded grants not resulting in publication and (2) the proportion of funded grants resulting in publication but not reporting data sufficient for meta-analysis. These proportions provide a direct assessment of the extent of study publication bias. In cases of non-publication, we contacted the investigators to request the unpublished data and to ask why they had not been published.

Two raters independently extracted data. Boolean formulas in Excel were used to compare and flag any mismatches between the two sets of data extractions. Discrepancies were resolved by consensus after checking the primary data. We calculated post-treatment effect sizes for four different types of comparisons: (1) psychological treatment versus control conditions, (2) psychological treatment versus another psychological treatment, (3) psychological treatment versus antidepressant medication, and (4) psychological treatment combined with antidepressant medication versus medication alone. We distinguished between no-treatment control conditions and control conditions in which some kind of treatment was provided; we subclassified treatment control conditions into (a) treatment-as-usual, (b) pill-placebo, and (c) psychological-placebo conditions intended to control for time and contact (nonspecific controls).

Within each study, effect size was calculated by subtracting the mean post-treatment score for one condition from the mean for the other, then dividing the difference by the pooled standard deviation, with positive signs indicating superiority of psychological treatment. For studies including multiple psychological treatment conditions, we ranked the different psychological treatment conditions in terms of the investigators’ presumed expected efficacy; effect sizes were then calculated in such a way that positive signs indicated superiority of the psychological treatment that we presumed the investigators expected to be more efficacious. This procedure is described in detail below.

If means and standard deviations were not reported, we used other data (e.g., event rates, reported effect sizes, *t-*statistics, or change scores, in that order) to compute the effect size. When no such data were available, the study was classified as not reporting sufficient data for meta-analysis. We calculated effect sizes as Hedges’ *g*, because it provides a less biased estimate than Cohen's *d* when sample sizes are small ([15], page 27). All meta-analyses were conducted using random effects models in Comprehensive Meta-analysis (version 2.2.064 [21]).

We used depressive severity as the sole outcome measure for this meta-analysis. Most commonly, depressive severity was measured with the Hamilton Depression Rating Scale [22] or the Beck Depression Inventory [23]. When a study’s results were reported separately for multiple outcome measures, a mean effect size was calculated and this synthetic score was then included in the meta-analysis ([15], pages 226–233). When a study’s results were reported for independent subgroups, we performed a fixed-effects meta-analysis on the subgroups to obtain a composite study-level effect size. These study-level effect sizes were then used in the meta-analysis ([15], pages 219–221). When a study included multiple versions of the same comparison (e.g., psychological treatment 1 versus waitlist control condition and psychological treatment 2 versus waitlist control condition), the mean effect size was calculated and this combined effect size for the study was included in the meta-analysis ([15], pages 239–241). Heterogeneity was examined using the Q and *I* ^*2*^ statistics. For the latter, we calculated the *I* ^*2*^ 95% confidence intervals [24] using the heterogi module [25] within Stata (Stata Statistical Software: Release 11), with the non-central chi-squared-based option [26].

In order to contrast the mean effect of the published studies with the mean effect of the unpublished studies, we conducted subgroup analysis testing using Comprehensive Meta-analysis (version 2.2.064 [21]). This employs a fully random effects analysis and pools study-to-study variance across subgroups ([15], pages 171–178), as recommended when subgroups involve small numbers of studies ([15], page 163). The pooled mean effect sizes were contrasted by means of a Q-test based on analysis of variance ([15], pages 177–178). If the p-value for the Q-between value (the weighted sum of squares of the subgroup means around the grand mean) is <.05, the pooled mean effect sizes of the two subgroups differ significantly, suggesting that effect size depends on publication status. Finally, we calculated the overall pooled mean effect size by combining published and unpublished studies. We calculated the change between this overall pooled mean effect size point estimate and the pooled mean effect size point estimate for published studies only (both the raw difference in Hedges’ g and the percentage change) using 5 decimals.

### Assigning ranks to comparisons of two different psychological treatments

For studies including multiple psychological treatment conditions, we ranked the different psychological treatment conditions in terms of the investigators’ presumed expected efficacy. Unlike other comparisons, such as psychological treatment versus control conditions (which can be assigned a positive sign if the psychological treatment is superior to the control condition and a negative sign if the control condition is superior to the psychological treatment), comparisons between different psychological treatments have no natural order. (For example, a hypothetical effect size comparing cognitive therapy with interpersonal therapy could be either -0.20 or +0.20.) Strategies used in earlier quantitative reviews to handle this matter include making all signs positive, which overestimates the pooled mean effect size, or assigning signs at random, which artificially biases the pooled mean effect size toward zero [27]. Neither approach was suitable for our purposes. Publication bias might be more likely if the treatment the investigators hypothesized to be more efficacious proved to be inferior to the treatment hypothesized to be less efficacious. Therefore, positive effect size values indicate superiority of the psychological treatment that the investigators expected, in our opinion (see below), to be more efficacious, while negative signs indicated superiority of the psychological treatment that the investigators expected to be less efficacious. If publication bias were present in comparisons of different psychological treatments, we hoped to be able to show it by this method, since we maximized the difference in effect sizes between published comparisons (likely positive since the psychological treatment expected to be more efficacious was superior) and unpublished comparisons (likely negative since the psychological treatment expected to be more efficacious was inferior).

We based our rankings of expected efficacy among the treatments on explicit statements to that effect whenever possible. If such statements were not evident, we based our rankings on the order of presentation of the respective treatments in the title or method section (in that order). In the case of dismantling studies (studies seeking to isolate separate components of a larger psychological treatment package), conditions including all components were ranked highest in terms of expected efficacy, while conditions with fewer components were ranker lower. Two authors judged presumed expected efficacy independently and disagreements were resolved by consensus. When consensus could not be reached, sensitivity analyses were conducted for each of the different rankings.

### Quality assessment of the included studies

The following four criteria of the Cochrane ‘risk of bias’ assessment tool were used to assess the quality of the included studies:

1. Intention-to-treat analysis (ITT): The study reports that ITT analyses have been conducted (whether or not the reported data used for effect size calculations include the total population).
2. Blind assessment of outcome: The outcome assessors were blinded and did not know to which condition the respondents were assigned. If only self-report measures were used to examine the effects, the study was coded as “no blind assessment”.
3. Adequate sequence generation: The investigators describe a random component in the sequence generation process.
4. Independent randomization: It is reported that randomization to conditions was conducted by an independent (third) party, with options including an independent person who conducted randomization or when a computer program or sealed envelopes were used to assign patients to conditions.

Unless the published article explicitly reported that a specific criterion had been met, we scored it as if it had not. We know from our communications with the investigators that these criteria were sometimes followed but not reported (on the assumption that they were standard methods that did not need to be made explicit) but we wanted to be consistent with other reviews that might be conducted solely on the basis of the published articles. Unpublished papers were rated from draft manuscripts whenever possible. When no drafts were available, the principal investigators of the unpublished studies were asked for quality criteria by email. Two raters independently rated these criteria, and disagreements were resolved by consensus.

## Results

**Fig 1:**
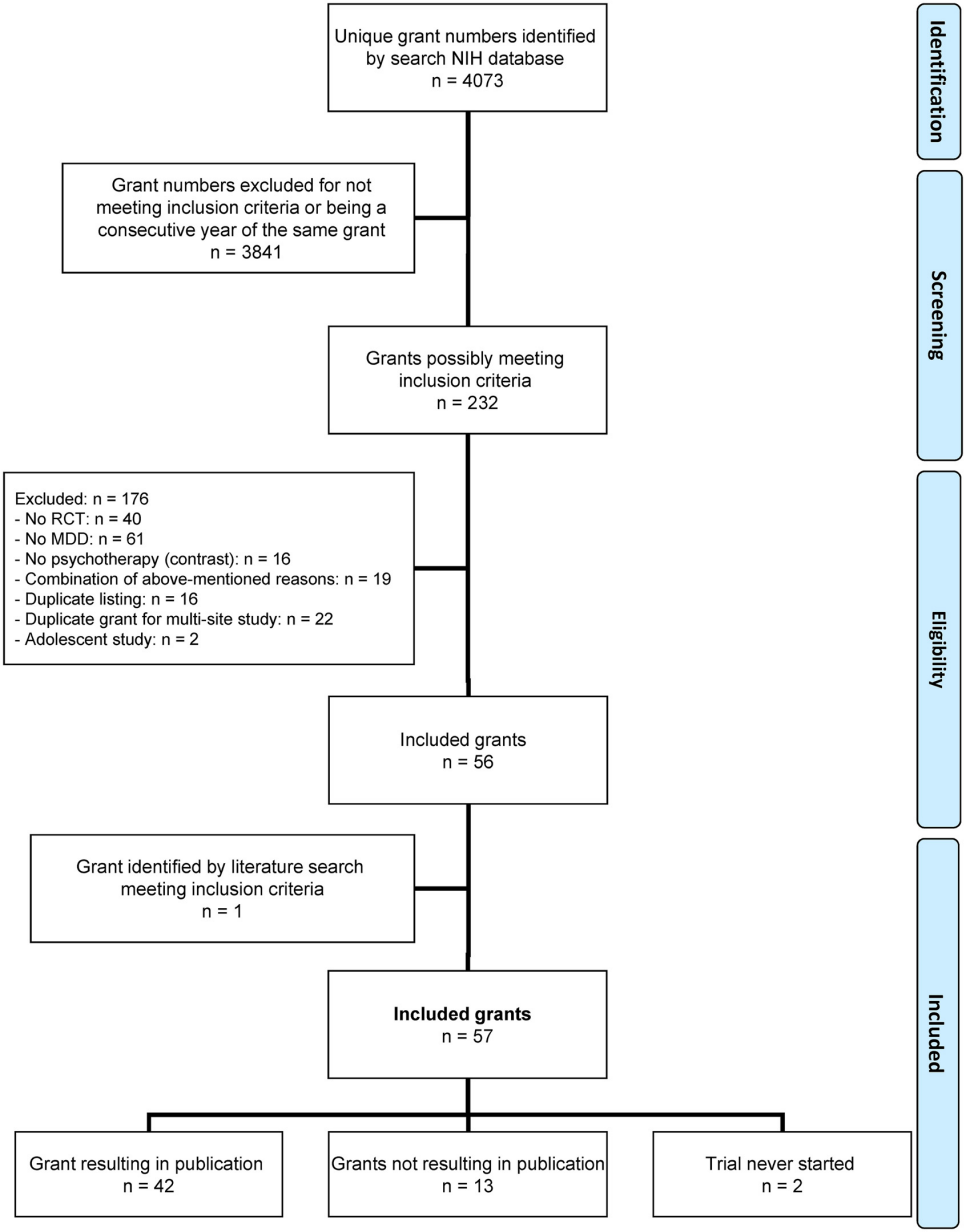
PRISMA flow chart of grants identified and the extent to which these led to publications. Note: MDD = major depressive disorder; NIH = US National Institutes of Health; RCT = randomized clinical trial.

As shown in Fig 1, 4073 grants were identified, of which 3841 were excluded based on titles and abstracts as not meeting inclusion criteria or as consecutive years of the same grant. Of the remaining 232 grants, 176 were excluded after a review of the published article or contacting the investigator, most often because the study population did not meet diagnostic criteria for MDD (n = 61). This left 56 grants that met our inclusion criteria.

We examined the completeness of our grant search strategy by checking all the published studies in the above-mentioned database for NIH grant acknowledgements. This yielded 38 articles that acknowledged NIH support but were not discovered in our search of the NIH grants database. In 13 of these 38 articles, the study participants were not selected on the basis of MDD; in another 5 studies all patients received the same psychological treatment. Three of the publications acknowledged NIH support that was not directly used to fund the study. One study was funded by a grant from the military and fifteen studies were funded by NIH grants awarded outside our cut-off years. In all, only one of the above-mentioned 38 articles met our inclusion criteria. That article described the results of an acute treatment trial [28] that had not been mentioned in the abstract of the NIH grant that provided its support; the original award listed only a maintenance trial and thus fell outside of the criteria for our database search. Because the acute trial met our inclusion criteria, we included the grant in our dataset, bringing the total number to 57.

### Extent of study publication bias

The 57 grants awarded are described in Table 1. Multiple grants that funded the same study are counted as a single grant, as are each of two grants that generated multiple published randomized clinical trials. Two other grant-funded studies were never started, one because of difficulty recruiting patients (S. Chisholm-Stockard, personal communication, March 3, 2011) and the other because of difficulty finding psychodynamic therapists willing to participate in clinical research with Hispanic elders (J. Szapocznik, personal communication, August 30, 2010). These last two grants were excluded from further consideration.

**Table 1:** Characteristics of the included grants.

	PI	Grant number	Ref.	Study comparison(s)	PT type(s)	Quality rating^a^			
	PUBLISHED					ITT	Blinding	Seq. gen.	Indep. Rand.
1	Arean	R01MH063982	[29]	PT vs CTRL-NS	PST	1	1	1	1
	Alexopoulos	R01MH064099							
2	Barber	R01MH061410	[30]	PT vs ADM	STPP	1	1	1	0
				PT vs CTRL-PLAC					
3	Beck	R01MH019989	[31]	PT vs ADM	CT	1	0	0	0
	Rush	R03MH027759							
4	Beutler	R01MH039859	[32]	PT vs CTRL-NS	CT	1	0	0	0
				PT vs PT	FEP				
5	Covi	R01MH033585	[33]	PT vs PT	CBT	0	0	0	0
					IPP				
6	Denton	K23MH063994	[34]	PT+ADM vs ADM	EFT	1	1	1	1
7	DeRubeis	R10MH055877^b^	[35]	PT vs ADM	CT	1	1	0	0
	Hollon	R10MH055875		PT vs CTRL-PLAC					
	Hollon	K02MH001697							
8	DiMascio	R01MH026467	[36–37]	PT vs ADM	IPT	0	1	0	0
	Weissman	R01MH026466		PT vs CTRL-NS					
				PT+ADM vs ADM					
9	Frank	R01MH065376	[38]	PT vs ADM	IPT	1	1	0	0
10	Frank	R21MH061948	[39]	PT vs PT	IPT	1	1	1	1
					IPT-PS				
11	Freedland	R21MH052629	[40]	PT+CTRL-NS vs CTRL-NS	CBT	1	0	1	1
12	Glick	R01MH034466	[41–42]	PT+CTRL-TAU+ADM vs CTRL-TAU+ADM	IFI	0	1	0	0
13	Greenberg	R01MH045040	[43]	PT vs PT	CCT	0	0	0	0
					EFT				
14	Hersen	R01MH028279	[44]	PT+ADM vs ADM	SST	0	1	0	0
				PT+CTRL-PLAC vs PT+CTRL-PLAC	STPP				
15	Hollon	R01MH033209	[45]	PT vs ADM	CT	1	1	0	0
				PT+ADM vs ADM					
16	Hollon	R01MH060713	[46]	PT+ADM vs ADM	CT	1	1	1	1
	Fawcett	R01MH060768							
	DeRubeis	R01MH060998							
17	Imber	U01MH033753	[47]	PT vs ADM	CBT	1	1	1	1
	Watkins	U01MH033760		PT vs CTRL-PLAC	IPT				
	Sotsky	U01MH033762		PT vs PT					
18	Jacobson	R01MH033838	[48]	PT vs PT	CBT	0	0	0	0
					BMT				
					CO				
19	Jacobson	R01MH055502	[49]	PT vs ADM	CT	1	1	1	0
				PT vs CTRL-PLAC	BA				
				PT vs PT					
20	Jacobson	R37MH044063	[50]	PT+PT+PT vs PT+PT	CT	0	0	0	0
				PT+PT+PT vs PT	P-CT				
				PT+PT vs PT	BA				
21	Jarrett	R01MH045043	[51]	PT vs ADM	CT	1	1	1	1
				PT vs CTRL-PLAC					
22	Keefe	R01NS046422	[52]	PT vs CTRL-NS	SeST	0	1	0	0
				PT vs CTRL-TAU					
23	Lustman	R01DK036452	[53]	PT vs CTRL-NS	CBT	0	0	1	0
24	Lynch	R03MH057799	[54]	PT+ADM vs ADM	DBT	0	0	0	0
25	Manber	R21MH066131	[55]	PT+ADM vs CTRL-NS+ADM	CBTI	0	1	1	0
26	Miller	R01MH035945	[56]	CTRL-TAU+ADM+PT vs CTRL-TAU+ADM	CT	0	0	0	0
				CTRL-TAU+ADM+PT vs CTRL-TAU+ADM+PT	SST				
27	Miranda	R01MH056864	[57]	PT vs ADM	CBT	1	1	1	1
				PT vs CTRL-TAU					
28	Mohr	R01MH059708	[58]	PT vs ADM	CBT	1	0	0	0
				PT vs PT					
29	Murphy	R01MH032756	[59]	PT vs ADM	CT	1	0	1	1
				PT+ADM vs ADM					
			[60]	PT vs ADM	CBT	0	0	1	0
				PT vs CTRL-NS					
30	O’Hara	R01MH050524	[61]	PT vs CTRL-NT	IPT	1	0	1	0
31	Reynolds	R01MH37869	[28]	PT+ADM vs ADM	IPT	1	1	0	0
				PT+CTRL-PLAC vs CTRL-PLAC					
32	Rohan	R03MH065946	[62]	PT vs CTRL-NT	CBT	1	1	1	1
33	Schulberg	R01MH045815^c^	[63]	PT vs ADM	IPT	1	1	0	0
				PT vs CTRL-TAU					
34	Simon	R01MH068127	[64]	PT+CTRL-TAU vs CTRL-TAU	CBT	1	0	1	1
35	Spinelli	K20MH001276	[65]	PT vs CTRL-NS	IPT	1	0	0	0
36	Swartz	K23MH064518	[66]	PT1 vs CTRL-TAU	IPT-MOMS	1	1	0	0
37	Talbot	K23MH064528	[67]	PT1+CTRL-TAU vs CTRL-TAU	IPT	1	0	0	0
38	Taylor	M01RR000070	[68]	PT vs CTRL-NT	CBT	1	1	0	0
39	Thompson	R01MH032157	[69]	PT vs PT	BT	0	1	0	0
					CT				
					STPP				
40	Thompson	R01MH037196	[70]	PT vs CTRL-NT	BT	0	0	0	0
				PT vs PT	CT				
			[71]	PT vs ADM	STPP	1	0	0	0
				PT+ADM vs ADM	CBT				
41	Weissman	R01MH034501	[72]	PT vs PT	IPT	0	1	0	0
					IPT-CM				
42	Wright	R21MH057470	[73]	PT vs CTRL-NT	CT	1	1	0	0
				PT vs PT	C-CT				
	**UNPUBLISHED**								
43	Battle	K23MH066402		PT vs CTRL-NT	IFT	1	0	1	1
44	Blum	R01MH025258		PT vs CTRL-NT	STPP	0	0	0	0
				PT vs PT	TOP				
45	Chisholm	F32MH012228*							
46	Clark	R01MH062054^d^							
47	Delgado	R01MH048977		PT vs ADM	CT	-	-	-	-
48	Gilliam	R01NS040808		PT vs ADM	CBT	0	0	1	1
49	Gottlieb	K07MH000597		PT vs ADM	CBT	-	-	-	-
				PT+ADM vs ADM					
50	Hauenstein	R18MH049101		PT vs CTRL-NS	CBT	0	1	1	1
51	Miller	R01MH058866		PT+ADM vs ADM	CT	1	1	1	1
				PT+PT+ADM vs PT+ADM	FT				
52	Monk	R34MH072838		PT vs CTRL-TAU	IPT	0	1	1	1
53	Stuart	R01MH059103		PT vs CTRL-TAU	IPT	-	-	-	-
54	Stuart	R01MH059668		PT vs PT	S-IPT	1	1	0	0
					CM-IPT				
55	Szapocznik	R01MH037379*							
56	Thase	R01MH041884		PT vs ADM	CBT	1	1	1	1
				PT vs CTRL-PLAC					
57	Zlotnick	R21MH060216		PT+ADM vs ADM	SFT	1	0	1	1

* Trial was never started.

^a^ 0 = not meeting quality criterion; 1 = meeting quality criterion

^b^ Grant number reported incorrectly in the published article, but confirmed with authors

^c^ Grant number omitted in published article but confirmed with authors

^d^ Investigator refuses to share data for this review.

ADM = Antidepressant medication; BA = behavioral activation; BMT = behavioral marital therapy; BT = behavior therapy; CBT = cognitive behavioral therapy; CBTI = cognitive behavioral therapy for insomnia; CCT = client-centered therapy; C-CT = computer-assisted cognitive therapy; CM-IPT = clinician-managed interpersonal psychotherapy; CO = combined treatment of BMT and CT; CP = counseling psychotherapy; CT = cognitive therapy; CTRL-NS = non-specific (psychological placebo) control condition; CTRL-PLAC = pill-placebo control condition; CTRL-NT = no-treatment control condition; CTRL-TAU = treatment-as-usual control condition; DBT = dialectical behavior therapy; EFT = emotion(ally)-focused therapy; FEP = focused expressive psychotherapy; FT = family therapy; IFI = inpatient family intervention; IFT = immediate family treatment; Indep. Rand. = independent randomization; IPP = interpersonal psychodynamic psychotherapy; IPT = interpersonal psychotherapy; IPT-CM = conjoint marital interpersonal psychotherapy; IPT-MOMS = interpersonal psychotherapy for depressed mothers; IPT-PS = interpersonal psychotherapy for depression and co-occurring panic symptoms; ITT = Intention to treat analysis; P-CT = partial cognitive therapy; PI = principle investigator; PST = problem-solving treatment; PT = psychological treatment; Ref. = reference number; Seq. gen = sequence generation; SeST = self system therapy; SFT = schema-focused therapy; S-IPT = standard interpersonal psychotherapy; SST = social skills training; STPP = short-term psychodynamic/psychoanalytic psychotherapy, TOP = theme-oriented psychotherapy.

Of the 55 grants that started studies, we were able to locate published articles corresponding to 42 (76.4%) grants [28–73], but not for the other 13 (23.6%). (If the two studies that weren’t started had not been excluded from consideration, the proportion of grants that did and did not lead to publications would have been 73.7% (42/57) and 26.3% (15/57), respectively). These 13 grants met our definition of unpublished studies. We were able to obtain the original data from 11 of these studies (84,6%). With respect to the remaining studies, the twelfth investigator was not yet ready to share her data (R. Clark). The thirteenth investigator expressed willingness to share data, but the data had been collected over a quarter of a century earlier and had not been retained, though he recalled that the sample was small (no more than a dozen patients per condition) and that the differences were negligible (G. Gottlieb, personal communication, June 10, 2012). Therefore, we excluded this study from the main analyses, but conducted sensitivity analyses, including this study in the relevant comparisons (psychological treatment alone or in combination versus antidepressant medication monotherapy) and estimating the study’s effect size to be *g* = 0.00 with n = 10 per condition. The total number of participants over the 42 published studies (4581) and 11 unpublished studies for which we had data (839) was 5420.

All but one of the published studies reported outcome data sufficient for calculating effect size. The remaining study [39] reported the grant-funded trial’s outcomes only in aggregate with outcomes of other trials. We requested the data for the grant-funded trial from the principal investigator. We counted the grant as published, because it resulted in a publication, but also conducted a sensitivity analysis coding it as unpublished, which resulted in published and unpublished rates of 74.5% (41/55) and 25.5% (14/55), respectively.

### Adjusting for study publication bias

**Fig 2:**
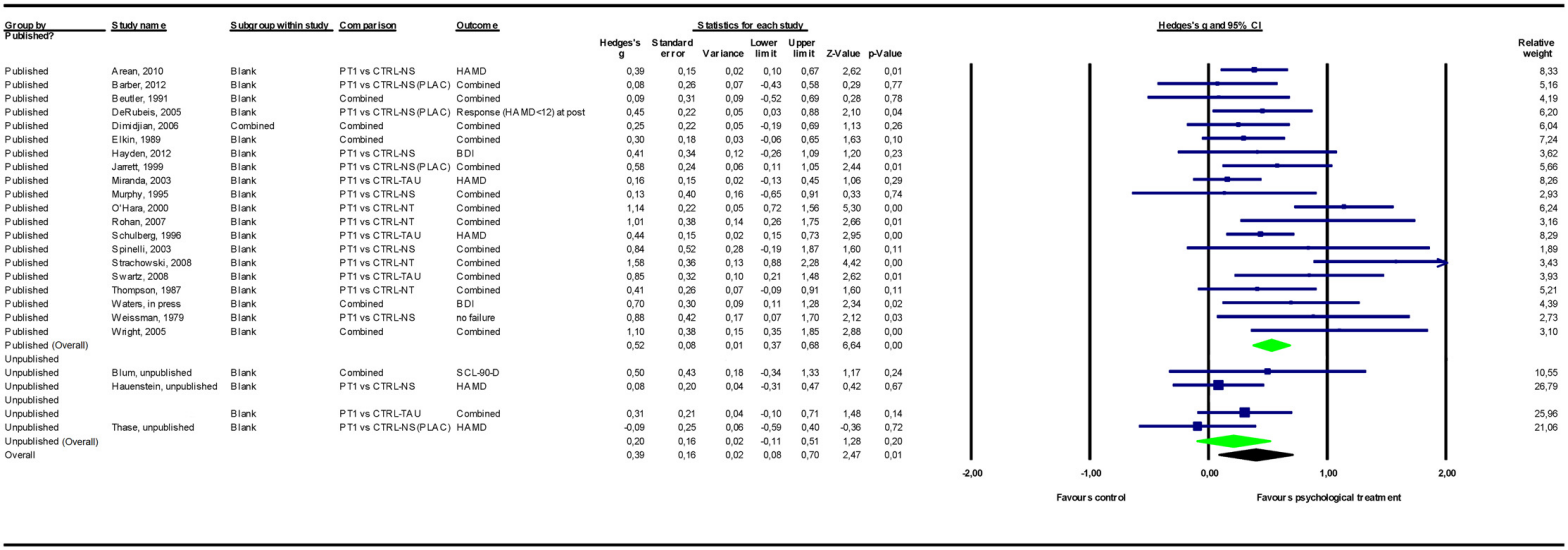
Psychological treatment versus control conditions (all). Note: Not all results of the unpublished studies are presented at study level, because we did not have permission of the investigators to do so. BDI = Beck Depression Inventory; CTRL-NS = non-specific control condition (psychological placebo); CTRL-NS(PLAC) = pill-placebo control condition; CTRL-NT = no-treatment control condition; CTRL-TAU = treatment as usual control condition; HAMD = Hamilton Depression Rating Scale; PT = psychological treatment; SCL-90-D = Symptom Checklist—90 item, depression subscale.

**Fig 3:**
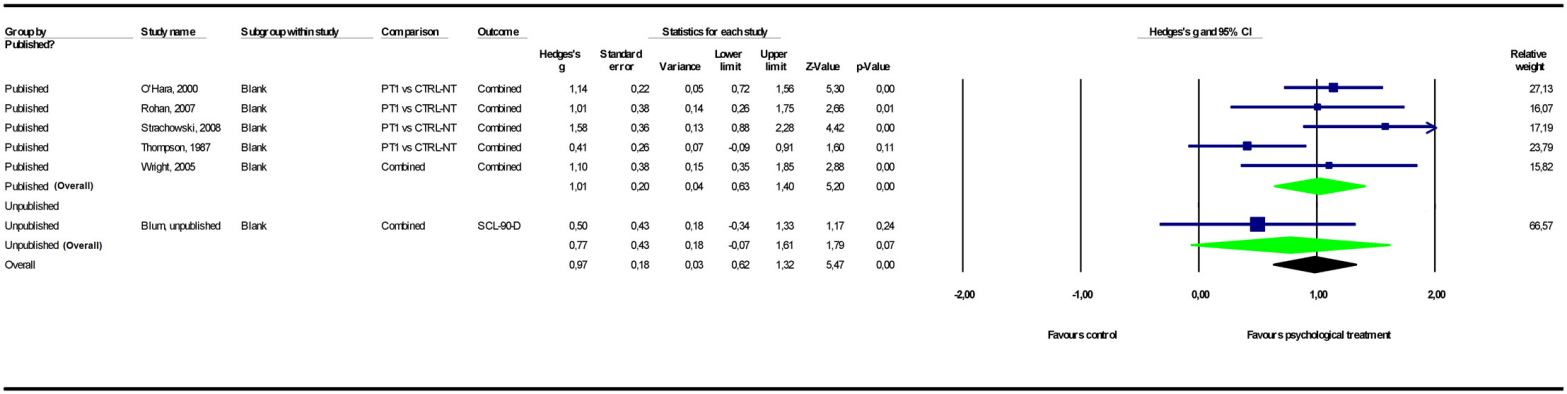
Psychological treatment versus no-treatment control conditions. Note: Not all results of the unpublished studies are presented at study level, because we did not have permission of the investigators to do so. CTRL-NT = no-treatment control condition; PT = psychological treatment; SCL-90-D = Symptom Checklist—90 item, depression subscale.

**Fig 4:**
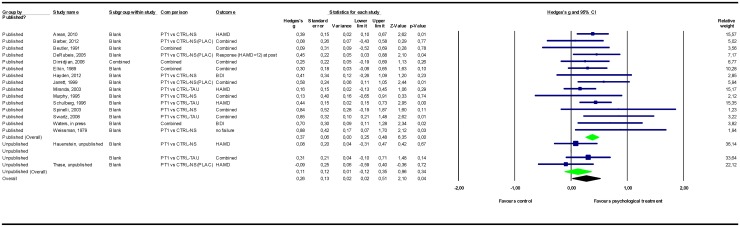
Psychological treatment versus treatment control conditions. Note: Not all results of the unpublished studies are presented at study level, because we did not have permission of the investigators to do so. BDI = Beck Depression Inventory; CTRL-NS = non-specific control condition (psychological placebo); CTRL-NS(PLAC) = pill-placebo control condition; CTRL-TAU = treatment as usual control condition; HAMD = Hamilton Depression Rating Scale; PT = psychological treatment.

**Fig 5:**
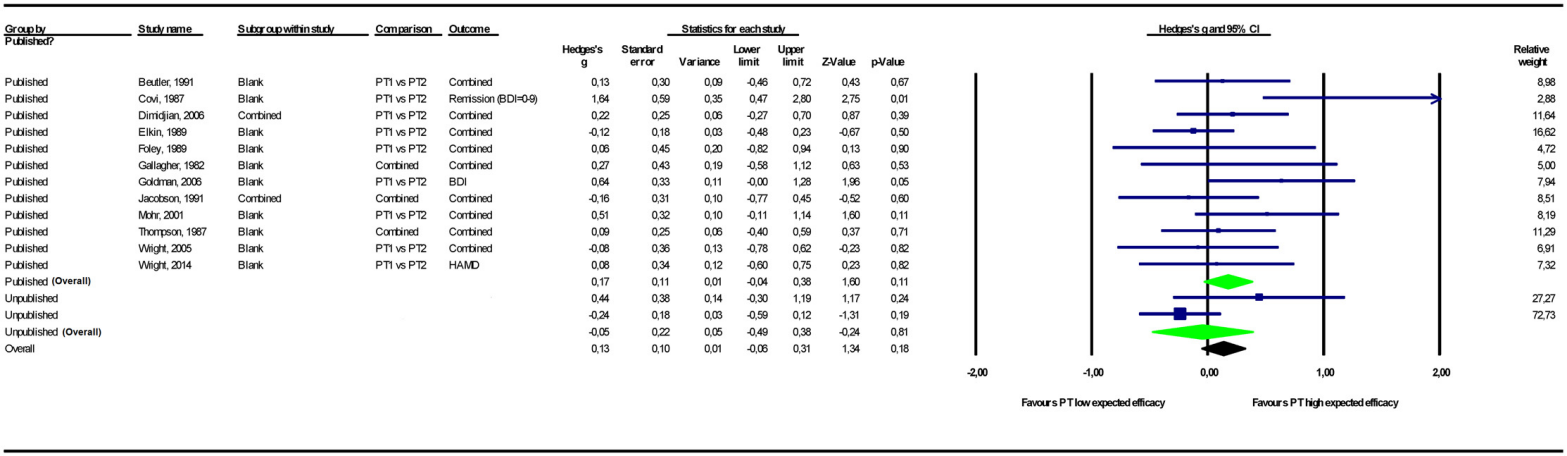
Psychological treatment versus other psychological treatment. Note: Not all results of the unpublished studies are presented at study level, because we did not have permission of the investigators to do so. BDI = Beck Depression Inventory; HAMD = Hamilton Depression Rating Scale; PT = psychological treatment.

**Fig 6:**
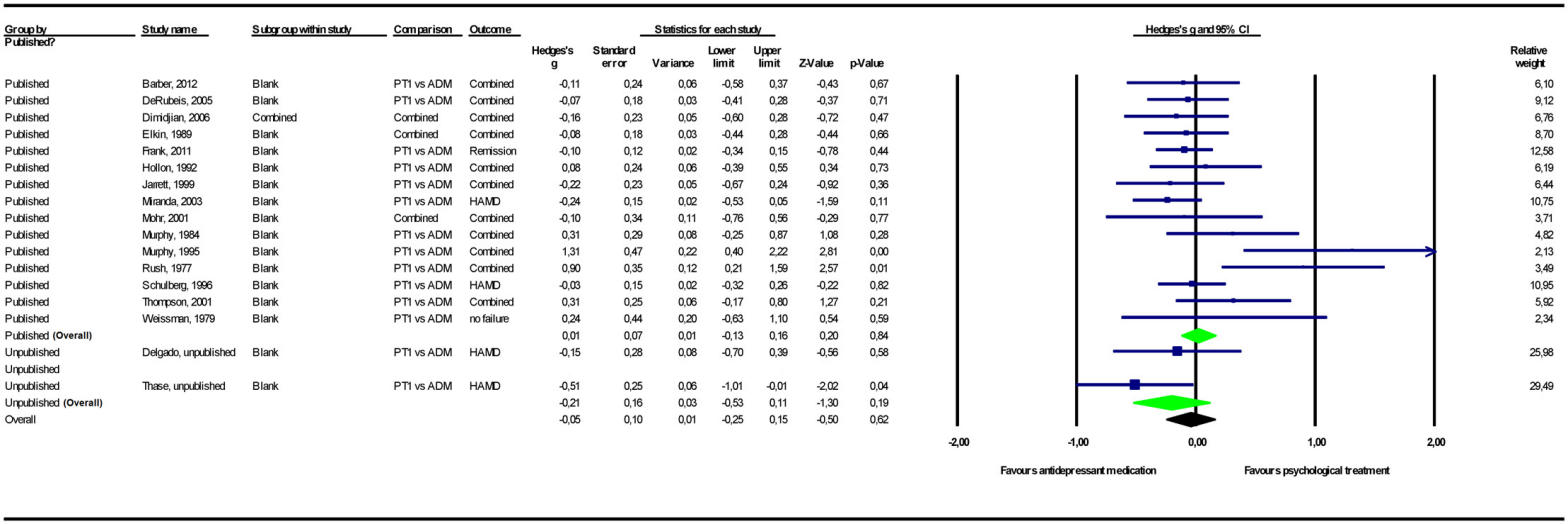
Psychological treatment versus antidepressant medication. Note: Not all results of the unpublished studies are presented at study level, because we did not have permission of the investigators to do so. ADM = antidepressant medication; HAMD = Hamilton Depression Rating Scale; PT = psychological treatment.

**Fig 7:**
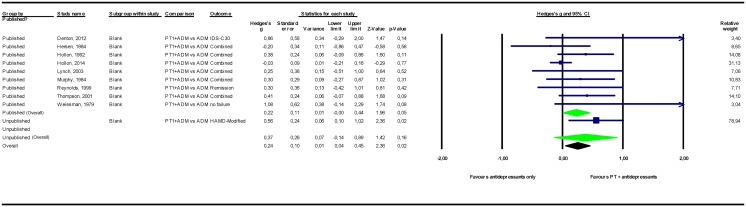
Psychological treatment combined with antidepressant medication versus antidepressant medication monotherapy. Note: Not all results of the unpublished studies are presented at study level, because we did not have permission of the investigators to do so. ADM = antidepressant medication; HAMD = Hamilton Depression Rating Scale; IDS-C30 = Inventory of Depressive Symptomatology– 30-item Clinician Rated version; PT = psychological treatment.

The results of the meta-analyses are presented in Table 2. Forest plots for these meta-analyses are provided in Figs 2–7.

**Table 2:** Meta-analyses of studies examining the effect of psychological treatment for depression.

	k	*g*	95% CI	*Z*	*Q*		*I* ^*2*^		Δg^a^		Q_betw_ ^b^	*p*	*N*_*PT*_	*N*_*Comp*_
					*Q*	*(df)*	*I* ^*2*^	*95% CI*	Δg	%				
**1. PT vs. Controls (all)**											3.34	.07		
Unpublished	6	0.20	-0.11~0.51	1.28	5.22	5	4	0~63					190	146
Published	20	0.52	0.37~0.68	6.64**	39.04**	19	51	7~70					959	808
Published + unpublished	26	0.39	0.08~0.70	2.47*	49.82**	25	50	12~67	-0.13	-25%			1149	954
1a. PT vs. no-treatment controls											0.28	.60		
Unpublished	2	0.77	-0.07~1.61	*1*.*79*	1.02	1	2	-					45	14
Published	5	1.01	0.63~1.40	5.20**	*8*.*39*	4	52	0~81					188	122
Published + unpublished	7	0.97	0.62~1.32	5.47**	9.88	6	39	0~73	-0.04	-4%			233	136
1b. PT vs. treatment controls											3.65	.06		
Unpublished	4	0.11	-0.12~0.35	0.96	1.59	3	0	0~68					145	132
Published	15	0.37	0.25~0.48	6.35**	11.83	14	0	0~46					771	686
Published + unpublished	19	0.26	0.02~0.51	2.10*	17.08	18	0	0~43	-0.11	-29%			916	818
**2. PT vs. other PT**											0.82	.37		
Unpublished	2	-0.05	-0.49~0.38	-0.24	2.61	1	62	-					78	86
Published	12	0.17	-0.04~0.38	1.60	13.74	11	20	0~59					286	331
Published + unpublished	14	0.13	-0.06~0.31	1.34	18.17	13	28	0~61	-0.04	-24%			364	417
**3. PT vs. antidepressant medication**											1.60	.21		
Unpublished	3	-0.21	-0.53~0.11	-1.30	2.24	2	11	0~76					116	115
Published	15	0.01	-0.13~0.16	0.20	*22*.*67*	14	38	0~65					840	835
Published + unpublished	18	-0.05	-0.25~0.15	-0.50	*26*.*39*	17	36	0~62	-0.07	-456%			956	950
**4. PT + antidepressant medication vs. medication only**											0.28	.59		
Unpublished	2	0.37	-0.14~0.89	1.42	2.20	1	54	-					41	44
Published	9	0.22	-0.00~0.44	*1*.*96*	10.63	8	25	0~65					389	423
Published + unpublished	11	0.24	0.04~0.45	2.36*	14.59	10	31	0~65	+0.02	+11%			430	467

* *p* < .05;

** *p* < .01; Italic numbers indicate a non-significant trend (*p* < .10)

^a^ difference between the effect size point estimates of the published studies only and the published + unpublished studies, calculated using 5 decimals.

^b^ 1 degree of freedom.

***N***_***Comp***_ = total number of participants in the comparisons conditions; ***N***_***PT***_ = total number of participants in the PT or PT+antidepressant medication conditions; PT = psychological treatment; **Q**_**betw**_. = Q between value

#### Psychological treatment versus control conditions

Among the studies that compared psychological treatments to control conditions (of any type), the overall pooled mean effect size was *g* = 0.20 (CI_95%_ -0.11~0.51) in the 6 unpublished studies (n = 336) versus *g* = 0.52 (0.37~0.68) in the 20 published studies (n = 1767). Adding unpublished studies to published studies resulted in a 25% decrease in effect size point estimate to *g* = 0.39 (0.08~0.70).

Among the different types of control conditions, when psychological treatment was compared to a no-treatment control condition, the pooled mean effect size in the 2 unpublished studies was *g* = 0.77 (-0.07~1.61) versus *g* = 1.01 (0.63~1.40) in the 5 published studies. Adding unpublished studies to published studies resulted in a 4% decrease in effect size point estimate to *g* = 0.97 (0.62~1.32). Comparing psychological treatments to treatment control conditions resulted in a pooled mean effect size of *g* = 0.11 (-0.12~0.35) for the 4 unpublished studies and *g* = 0.37 (0.25~0.48) for the 15 published studies. Adding unpublished to published studies resulted in a 29% decrease in effect size point estimate to *g* = 0.26 (0.02~0.51). For all the above-mentioned comparisons, psychological treatments remained significantly more efficacious than the control conditions after the unpublished data were added to the published data.

Among the different types of treatment control conditions (treatment-as-usual, pill-placebo, and non-specific [psychological placebo]), the largest differential between published and unpublished findings was apparent for comparisons of psychological treatment to pill-placebo (S1 Table). The pooled mean effect size of psychological treatment relative to pill-placebo control condition was *g* = -0.09 (-0.59~0.40) in 1 unpublished study and *g* = 0.34 (0.14~0.53) in 5 published studies. Adding unpublished studies to published studies resulted in a 45% decrease in effect size point estimate to *g* = 0.19 (-0.21~0.59).

#### Psychological treatment versus other psychological treatments

Two unpublished studies involving a total of 164 participants were found comparing different types of psychological treatments. The pooled mean effect size in these 2 studies was *g* = -0.05 (CI_95%_ -0.49~0.38), while the pooled mean effect size in the 12 published studies totaling 617 participants was *g* = 0.17 (-0.04~0.38). Adding the unpublished studies to the published studies resulted in a 24% decrease in effect size point estimate to *g* = 0.13 (-0.06~0.31), indicating no significant differences between the psychological treatments that the investigators expected, in our opinion, to be most efficacious, and those expected to be less efficacious. We conducted a sensitivity analysis for one study intended to minimize allegiance effects [47]. Changing the psychological treatment ranks did not alter the pattern of results (S1 Table). We also conducted a sensitivity analysis for the one study that did not report the data required to analyze the results in a meta-analysis [39]. Counting this study as unpublished instead of published did not alter the pattern of results (S1 Table).

#### Psychological treatment versus antidepressant medication

When psychological treatment was compared to antidepressants, the pooled mean effect size was *g* = -0.21 (CI_95%_ -0.53~0.11) for 3 unpublished studies (n = 231) and *g* = 0.01 (-0.13~0.16) for 15 published studies (n = 1675). Adding unpublished to published studies resulted in a pooled mean effect size of *g* = -0.05 (-0.25~0.15), indicating no significant difference between psychological treatments and antidepressants. This pattern of results was not altered when we included the estimates from the unpublished study (Gottlieb) for which the original data were not available (S1 Table).

#### Combined psychological treatment and antidepressants versus antidepressant monotherapy

The pooled mean effect size of combined treatment in which psychological treatment was added to antidepressant medication versus medication monotherapy was *g* = 0.37 (CI_95%_ -0.14~0.89) for 2 unpublished studies (n = 85) and *g* = 0.22 (-0.00~0.44) for 9 published studies (n = 812). Adding unpublished to published studies resulted in an effect size point estimate of *g* = 0.24 (0.04~0.45), indicating that combined treatment was superior to antidepressant medications alone. Including the estimates from one unpublished study for which the original data were not available (Gottlieb) did not alter this result pattern (S1 Table).

### Reasons for non-publication

Of the 13 unpublished studies, only two were submitted for review; neither was accepted for publication. Six other studies were never submitted for review, although, in three instances, the investigators still hoped to do so. For the remaining five studies, it was unclear whether the authors tried to submit their findings for publication. Explanations that the investigators gave for not submitting manuscripts included that they did not think the findings were interesting enough to warrant publication, that they got distracted by other obligations or that they had practical problems.

### Quality of the included studies

We assessed the quality of the included studies (see Table 1) with regard to the four quality criteria. We were not able to retrieve quality ratings for three of the unpublished studies. Study quality was less than optimal; only 10 of the 53 (18.9%) studies were rated ‘low risk of bias’ on all four criteria. No significant difference was found in the proportion of such studies between the published (18.2%) and unpublished (22.2%) studies (Fisher’s Exact test, *p* = 1.00). Similarly, no significant difference was found between the published and unpublished studies with regard to the proportion of studies that reported intention-to-treat analyses (respectively, 63.6% and 55.6%, Fisher’s Exact test, *p* = .72) or that kept outcome assessors blind to treatment allocation (respectively, 56.8% and 55.6%, Fisher’s Exact test, *p* = 1.00). However, the number of studies that reported an adequate sequence generation evidenced a nonsignificant trend (38.6% and 77.8%, Fisher’s Exact test, *p* = .06) and the number of studies that reported that an independent party conducted the randomization was significantly smaller among the published studies (25.0%) than among the unpublished studies (77.8%; Fisher’s Exact test, *p* = .005). This pattern of findings did not change when we recoded the three unpublished studies with missing quality ratings as high risk of bias on all four criteria.

We also tested whether the mean number of participants per treatment condition differed between unpublished (31.9, SD = 22.2) and published (41.9, SD = 42.5) studies. Only about 75% as many patients were used per condition in the unpublished studies compared to the published studies, but this difference was not significant (*t*(54) = 0.79, p = .44).

## Discussion

We found clear indications of study publication bias in NIH-funded randomized clinical trials examining the efficacy of psychological treatment for major depressive disorder; 23.6% (13/55) of the grant-funded studies did not result in a publication. The binomial 95% confidence interval of this proportion (13%-37%) overlaps considerably with that of the 31.1% (23/74; binomial confidence interval 21%-43%) non-publication rate reported by Turner and colleagues for industry-funded trials of antidepressant medications [10].

When the unpublished findings were added to the published findings for comparisons of psychological treatments vis-à-vis control conditions (in aggregate), the effect size point estimate was reduced 0.13 standard deviations (from *g* = 0.52 to *g* = 0.39). Using data from the earlier study by Turner and colleagues [10] showed that the effect size for antidepressant medications should be adjusted downward by 0.07 standard deviations when unpublished findings were added those that had been published. Our findings are based on different sets of trials than Turner’s and we can have less confidence in our estimate for psychotherapy since it was based on a smaller sample. Our findings indicate that psychological treatment is efficacious and specific, but, as is the case for antidepressants, less than the published literature conveys.

Our findings are in line with previous studies that suggest the presence of publication bias in studies of psychotherapy for depression [11, 74]. However, in contrast to those studies, which relied on an inferred estimate based on statistical procedures, this study ascertains the extent of study publication bias through direct empirical assessment. By tracking a cohort of US NIH-funded grants, we were able to determine how many trials went unpublished. It is reassuring that all but one of the published studies in our sample reported data in a manner suitable for meta-analysis and that, for the unpublished studies, there was a general willingness to share data.

A major limitation of this study is that we were not able to ascertain the extent to which the published results might have been affected by outcome reporting bias, thus exaggerating their effects. Unfortunately, there is no equivalent to the FDA database that would allow us to do for psychological treatment studies what Turner and colleagues were able to do with antidepressant trials [10], namely to determine whether, and quantify the degree to which, the published results had been “spun”. We tried to obtain the original grants from the NIH, but only a handful of the earlier protocols (before 2000) had been retained. This contrasts with the success of Chan and colleagues in obtaining protocols from the Canadian Institutes of Health Research [75]. If we had had similar access to the NIH protocols, we could have ascertained the degree to which the authors adhered to the *a priori* methods when generating the corresponding publications. We believe that the NIH should archive protocols and make them publicly available so that outcome reporting bias can be ruled out by researchers and discouraged among investigators.

However, even with full access to the protocols, we still would not know whether the results reported were obtained using the methods prespecified *a priori* in the protocols. Therefore, we cannot say to what extent our effect sizes (adjusted for study publication bias alone) represent the ‘true’ effect of psychological treatment. If we could have detected outcome reporting bias, the adjusted effect would probably have been lower than what we report after adjusting for study publication bias alone. In essence, we can never come to terms with the extent of publication bias in the literature unless we can correct for both study publication bias and outcome reporting bias. The field needs both a clinical trials registry and a data repository for psychological treatment trials similar to the one the FDA maintains for industry-funded pharmacotherapy trials.

This study also has several additional limitations. (1) We were not able to obtain unpublished data for 2 of the 13 (15.4%) unpublished studies. In one case, the investigator was willing, but data collected in the 1980s were no longer available. However, in the other case, the investigator was reluctant to share her data, fearing that doing so would jeopardize her chances for independent publication. (2) Although unlikely, our search might have missed grant-funded randomized clinical trials that were begun but never published. We did find one instance in which we had excluded a grant that resulted in a publication that fell within our criteria. We suspect that this was a rare occurrence that came about only because the sample studied was available for inclusion in a separate acute treatment trial after it was excluded from the funded maintenance trial [76]. (3) Our sample was restricted to studies funded by grants from the US government. Studies conducted in other countries or funded by other sources could show different characteristics and publication patterns. (4) No review protocol was registered for this study. When we started our review in 2009, registration of protocols for reviews was uncommon [77]; the international prospective register of systematic review protocols in health and social care (PROSPERO) was launched in February 2011, by which time our review was well underway. Nevertheless, review protocol registration was possible [77] and, in retrospect, would have increased the transparency of our study by allowing readers to compare our published methods with those originally planned. (5) The quality of the studies included in this meta-analysis was less than optimal. (6) Many of the analyses included only a small number of unpublished studies. (7) Combining different types of psychological treatments or different types of comparison conditions might have increased heterogeneity. (8) Given that we used depressive symptoms as the sole outcome for this review, it is possible that we might have missed studies that excluded such measures,but all published and unpublished studies did include such measures, such that no study was excluded from the analyses for this reason. (9) It is possible that we missed published articles; however, we think that is unlikely because we searched a comprehensive database of published randomized clinical trials of psychotherapy for depression, conducted a literature search for all publications by the grant’s principal investigator, then, when we could not locate a published study, contacted the investigators. (10) Our examination of the completeness of our grant search strategy, which involved checking for grant acknowledgements within all published studies listed in a database, could be criticized for introducing bias, as it could only result in the inclusion of additional published studies and not unpublished studies. However, excluding the one study that was added to the sample as a consequence of this examination from the analyses did not affect the general pattern of results. (11) Finally, some of the unpublished psychological treatment studies may still be published in the future. The investigators of two of the more recent trials that obtained relatively large effect sizes indicated that they still intended to submit their studies. If these were to be accepted that would lower the non-publication rate from 23.6% to 20%. It would not alter the “true” effect of psychotherapy based on all initiated trials; it would, however, widen the gap in effect size between the published and unpublished trials.

Most of the unpublished studies were not submitted for review. That is not to say that journals do not play a role in shaping author expectations, but the proximal cause of non-publication in our sample was more often non-submission by the authors rather than rejection by the journals. This is consistent with an earlier study of US NIH-funded clinical trials that found that findings were often not submitted when the investigators considered their results "not interesting" or when they “did not have enough time” [78]. At the same time, we doubt that the authors were trying to suppress less than impressive findings. We would speculate that most research clinicians would benefit more from a publication than they would be adversely affected by null findings. Nonetheless, the bias against null findings is so ingrained in the field that we doubt that simple exhortations to authors or editors will do enough to change behavior. Instead, we join with others who recommend that funding agencies or journals should archive both original protocols and raw data from any funded randomized clinical trial [79–83].

In addition, we believe that a loophole should be closed in the NIH Public Access Policy (http://publicaccess.nih.gov/policy.htm). This policy requires that results from NIH-sponsored studies be made freely available to the public (via open access publication), but it applies only if the trial results are published in peer-reviewed journals. If an investigator chooses to leave NIH-sponsored study results unpublished, he or she is still free to do so. But we believe that an investigator who accepts public funds to conduct research has a fiduciary and ethical duty to provide a return on the public’s investment by making a good faith effort to publish. We therefore call upon the NIH to expand its Public Access Policy so that the public can have, full, not partial, access. The widespread belief that “negative” results are “not publishable” is no longer as true as it once was, because there are now journals committed to publishing study results without regard to their strength and direction, including Trials and PLOS ONE.

In conclusion, study publication bias is present in studies funded by US government grants to examine the efficacy of psychological treatment for depression, and this leads to an overestimation of its effects. The reason why is open to debate, but the fact that it occurs is not. Our findings indicate that psychological treatment is efficacious and specific, but, as is the case for antidepressants, less than the published literature conveys. Clinicians, guidelines developers, and decision makers should be aware of overestimated effects of the predominant treatments for major depressive disorder.

## Supporting Information

S1 Table Meta-analyses of studies examining the effect of psychological treatment for depression (Additional and sensitivity analyses). * *p* < .05; ** *p* < .01; Italic numbers indicate a non-significant trend (*p* < .10). ^a^ difference between the effect size point estimates of the published studies only and the published + unpublished studies, calculated using 5 decimals. ^b^ 1 degree of freedom. ^c^ Sensitivity analysis changing psychotherapy ranks in the published study, which was intended to minimize allegiance effects [47]. ^d^ Sensitivity analysis counting [39] that does not report effect size data as unpublished. ^e^ Sensitivity analysis including the unpublished Gottlieb study, for which the effect size was estimated to be zero because of missing data. Note: *N*_*St*_ = number of studies; PT = psychological treatment; Q_betw_. = Q between value.
(DOCX)
