# Supplementary material for: Does Publication Bias Inflate the Apparent Efficacy of Psychological Treatment for Major Depressive Disorder? A Systematic Review and Meta-Analysis of US National Institutes of Health-Funded Trials
Source: PLoS One. 2015 Sep 30;10(9):e0137864. doi: 10.1371/journal.pone.0137864 (PMC4589340; doi:10.1371/journal.pone.0137864)
Supplement: S1 Table — * p < .05; ** p < .01; Italic numbers indicate a non-significant trend (p < .10). a difference between the effect size point estimates of the published studies only and the published + unpublished studies, calculated using 5 decimals. b 1 degree of freedom. c Sensitivity analysis changing psychotherapy ranks in the published study, which was intended to minimize allegiance effects [47]. d Sensitivity analysis counting [39] that does not report effect size data as unpublished. e Sensitivity analysis including the unpublished Gottlieb study, for which the effect size was estimated to be zero because of missing data. Note: N St = number of studies; PT = psychological treatment; Qbetw. = Q between value. (DOCX) [file pone.0137864.s001.docx]

**S1 Table. Meta-analyses of studies examining the effect of psychological treatment for depression (Additional and sensitivity analyses)**

|  | **k** | ***g*** | **95% CI** | ***Z*** | ***Q*** |  | ***I^2^*** |  | **Δg^a^** |  | **Q_betw_^b^** | ***p*** |
| --- | --- | --- | --- | --- | --- | --- | --- | --- | --- | --- | --- | --- |
|  |  |  |  |  | *Q* | *(df)* | *I^2^* | *95% CI* | Δg | % |  |  |
| **1b PT vs. Treatment controls** | | |  |  |  |  |  |  |  |  |  |  |
| *1b-I PT vs. treatment as usual* | | | | |  |  |  |  |  |  | 0.51 | .47 |
| Unpublished | 2 | 0.24 | -0.19~0.66 | 1.09 | 0.36 | 1 | 0 | - |  |  |  |  |
| Published | 4 | 0.42 | 0.17~0.66 | 3.37** | 5.29 | 3 | 43 | 0~80 |  |  |  |  |
| Published + unpublished | 6 | 0.37 | 0.16~0.58 | 3.47** | 6.05 | 5 | 17 | 0~67 | -0.04 | -11% |  |  |
| *1b-II PT vs. placebo* | | |  |  |  |  |  |  |  |  | 2.48 | .12 |
| Unpublished | 1 | -0.09 | -0.59~0.40 | -0.36 | 0.00 | 0 | 0 | - |  |  |  |  |
| Published | 5 | 0.34 | 0.14~0.53 | 3.43** | 2.55 | 4 | 0 | 0~64 |  |  |  |  |
| Published + unpublished | 6 | 0.19 | -0.21~0.59 | 0.91 | 5.04 | 5 | 1 | 0~61 | -0.15 | -45% |  |  |
| *1b-III PT vs. non-specific treatment (psychological placebo)* | | | | |  |  |  |  |  |  | 2.36 | .12 |
| Unpublished | 1 | 0.08 | -0.31~0.47 | 0.42 | 0.00 | 0 | 0 | - |  |  |  |  |
| Published | 7 | 0.43 | 0.22~0.63 | 4.11** | 4.77 | 6 | 0 | 0~58 |  |  |  |  |
| Published + unpublished | 8 | 0.30 | -0.03~0.63 | *1.78* | 7.13 | 7 | 2 | 0~57 | -0.13 | -31% |  |  |
| **PT vs. other PT^c^** | | | | |  |  |  |  |  |  | 1.75 | .19 |
| Unpublished | 2 | -0.08 | -0.46~0.29 | -0.42 | 2.61 | 1 | 62 | - |  |  |  |  |
| Published | 12 | 0.20 | 0.01~0.39 | 2.11* | 11.27 | 11 | 2 | 0~59 |  |  |  |  |
| Published + unpublished | 14 | 0.11 | -0.15~0.37 | 0.82 | 16.60 | 13 | 22 | 0~58 | -0.09 | -46% |  |  |
| **PT vs. other PT^d^** |  |  |  |  |  |  |  |  |  |  | 0.78 | .38 |
| Unpublished | 3 | -0.02 | -0.40~0.36 | -0.10 | 2.86 | 2 | 30 | 0~80 |  |  |  |  |
| Published | 11 | 0.18 | -0.04~0.39 | 1.60 | 13.71 | 10 | 27 | 0~63 |  |  |  |  |
| Published + unpublished | 14 | 0.13 | -0.06~0.32 | 1.34 | 18.17 | 13 | 28 | 0~61 | -0.05 | -27% |  |  |
| **PT vs. antidepressant medication^e^** | | | | |  |  |  |  |  |  | 1.42 | .23 |
| Unpublished | 4 | -0.19 | -0.48~0.11 | -1.24 | 2.43 | 3 | 0 | 0~68 |  |  |  |  |
| Published | 15 | 0.01 | -0.13~0.15 | 0.16 | *22.67* | 14 | 38 | 0~65 |  |  |  |  |
| Published + unpublished | 19 | -0.04 | -0.22~0.13 | -0.49 | *26.40* | 18 | 32 | 0~60 | -0.05 | -474% |  |  |
| **PT + antidepressant medication vs. medication only^e^** | | | | |  |  |  |  |  |  | 0.08 | .77 |
| Unpublished | 3 | 0.29 | -0.15~0.73 | 1.28 | 2.96 | 2 | 32 | 0~81 |  |  |  |  |
| Published | 9 | 0.21 | 0.00~0.43 | 1.96* | 10.63 | 8 | 25 | 0~65 |  |  |  |  |
| Published + unpublished | 12 | 0.23 | 0.04~0.42 | 2.32* | 14.70 | 11 | 25 | 0~62 | +0.01 | +6% |  |  |
